# Polymorphism of 9p21.3 Locus Is Associated with 5-Year Survival in High-Risk Patients with Myocardial Infarction

**DOI:** 10.1371/journal.pone.0104635

**Published:** 2014-08-08

**Authors:** Anna Szpakowicz, Marek Kiliszek, Witold Pepinski, Ewa Waszkiewicz, Maria Franaszczyk, Małgorzata Skawronska, Rafal Ploski, Anna Niemcunowicz-Janica, Sławomir Dobrzycki, Grzegorz Opolski, Włodzimierz Jerzy Musial, Karol Adam Kaminski

**Affiliations:** 1 Department of Cardiology, Medical University of Bialystok, Bialystok, Poland; 2 Department of Cardiology, Medical University of Warsaw, Warsaw, Poland; 3 Department of Forensic Medicine, Medical University of Bialystok, Bialystok, Poland; 4 Laboratory of Molecular Biology, Institute of Cardiology, Warsaw, Poland; 5 Department of Medical Genetics, Medical University of Warsaw, Warsaw, Poland; 6 Department of Invasive Cardiology, Medical University of Bialystok, Bialystok, Poland; Johns Hopkins University, United States of America

## Abstract

**Objective:**

The rs10757278, rs1333049 and rs4977574 are single nucleotide polymorphisms (SNPs) of chromosome 9p21 locus associated with a prevalence of acute coronary syndromes (ACS). Reports concerning their association with long-term outcome after an ACS are equivocal. The aim of our study was to investigate the association of the 9p21.3 locus with 5-year overall mortality in patients with ST-elevation myocardial infarction (STEMI).

**Materials and methods:**

We performed a retrospective analysis of data collected prospectively in 2 independent registries of consecutive patients with STEMI (derivation and validation group). Genotyping was performed with the TaqMan method. The analyzed end-point was total mortality.

**Results:**

The derivation group comprised 589 patients: 25.3% female (n = 149), mean age 62.4±12.0 years, total 5-year mortality 16.6% (n = 98). When all the study group was analyzed, no significant differences in mortality were found between the genotypes. However, in high-risk patients (GRACE risk score ≥155 points, n = 238), homozygotes associated with higher risk for ACS had significantly better 5-year survival compared to other genotypes. The hazard ratio associated with the high-risk genotype (a homozygote of high risk for ACS or a heterozygote) was: HR = 2.2 (1.15–4.2) for the rs10757278 polymorphism, HR = 2.7 (95% CI 1.3–5.4) for the rs4977574 one and HR = 2.3 (1.2–4.5) for the rs1333049 one (Cox proportional hazards model). Survival analysis in the validation group (n = 365) showed a clear trend towards better prognosis in GG homozygotes of the rs10757278 SNP, which confirms our initial results (p = 0.09, log-rank test).

**Conclusions:**

The 9p21.3 locus is associated with 5-year mortality in high-risk patients with STEMI. The genotypes associated with higher risk for ACS show a protective effect in terms of further survival (instead of a deteriorating prognosis, as reported previously). This finding, due to the very high size of the effect, could potentially be applied to clinical practice, if appropriate methods are elaborated.

## Introduction

Several genome-wide association studies have shown a strong association between the chromosomal locus 9p21.3 and coronary artery disease (CAD) or myocardial infarction, [1]–[8]. The results have been further replicated in large-scale case-control studies [9]–[10]. The same locus was reported to give a significant genomic signal for other diseases, like type 2 diabetes [11]–[14], aortic or intracranial aneurysms, peripheral artery disease, or cancers [15]–[17].

There are several single nucleotide polymorphisms (SNPs) of the 9p21.3 locus associated with cardiovascular diseases; however, the functional link is still poorly understood. None of the SNPs is located within a protein coding region. The 9p21.3 locus contains only a sequence for an antisense RNA (ANRIL, CDKN2BAS). The nearby genes are coding cyclin-dependent kinases 2B and 2A (CDKN2A and CDKN2B) or methylthioadenosine phosphorylase (MTAP). The SNPs in the 9p21 locus affect the expression of ANRIL, which has been shown to modulate atherogenic pathways in vascular smooth muscle cells, including CDKN2A/B regulation [18]–[19]. The CDKN2B is potentially involved in the pathogenesis of atherosclerosis, while it is a downstream target for transforming growth factor beta, [20]–[21].

There is very strong evidence for association between the 9p21.3 locus and myocardial infarction (MI). However, data regarding its influence on further prognosis is equivocal. In the GRACE registry that was performed in Europe (n = 3247, patients with all forms of an acute coronary syndrome, 6 months of follow-up), the C allele of the rs1333049 polymorphism was independently associated with recurrent myocardial infarction or cardiac death [22]. No association with outcome was found in the population of the Post-Myocardial Infarction study (New Zealand, n = 816, median follow-up 9 years) or in Han Chinese patients with first ST-segment elevation myocardial infarction (STEMI, n = 520, median follow-up 29 months) [23]–[24]. On the contrary, there is an increasing number of reports with surprisingly improved prognosis in patients with established CAD, or after an acute coronary syndrome, who carry alleles associated with a higher risk of atherosclerosis (25–27). This paper refers to a study by A. Szpakowicz et al. that was previously published in Plos One and retracted due to genotyping errors [28]. In this version we outline the identified errors, provide data from an additional validation group supporting our new results, and update our conclusions.

There is a debate as to whether genetic testing may become a part of risk assessment in MI patients. Some authors claim that the influence of particular polymorphisms on prognosis does not exceed 15–30%, thus making a whole genetic analysis useless for clinical risk assessment. Their opinions, however, are based on genome wide association studies that are especially biased to recognize markers with limited influence. This fact does not mean that particular polymorphisms would not be useful for special populations, where they could identify high risk patients.

In the present study we aimed to investigate the association of the 9p21.3 locus with 5-year all-cause mortality of patients with myocardial infarction. We tested 3 previously described SNPs (rs10757278, rs1333049, rs4977574) as markers of this locus. A further goal of the study was selecting and characterizing patients who could gain the greatest benefit from genotyping. This paper corrects a previously published version of the article that was retracted due to genotyping errors [28].

## Materials and Methods

### Ethics statement

The study protocol was approved by the Ethics Committee of the Medical University of Bialystok. The study was performed in accordance with the ethical standards laid down in the 1964 Helsinki Declaration. Informed written consent has been obtained from all subjects.

We performed a retrospective analysis of data collected prospectively from 2 independent centers with a catheterization laboratory. It comprised Caucasian patients with STEMI who survived the first 48 hours after admission. Patients from the derivation group were inhabitants of North-Eastern Poland and were hospitalized in the years 2001–2005. Patients from the validation group came from central Poland (the Warsaw registry of acute coronary syndrome) and underwent the initial event in the years 2008–2010. The 48 hour survival threshold was implemented because of the time from blood sampling to the potential result of genetic testing. In this subpopulation it would be of no benefit, but the very early mortality might affect final results. No additional exclusion criteria were introduced. All the patients underwent coronary angiography within 12 hours of the onset of symptoms. STEMI was diagnosed based on a rise in troponin I concentrations or creatine kinase – MB fraction activity accompanied by chest pain history and new ECG abnormalities (ST-segment elevation or left bundle branch block lasting >20 minutes). The analyzed data included patients’ history, physical examination on admission, routine laboratory tests, echocardiography, results of coronary angiography, and invasive treatment. MDRD (Modification of Diet in Renal Disease) formula was used to estimate creatinine clearance. GRACE risk score was calculated retrospectively, based on a previously described method [29]. We used a version of the score that is recommended by the European Society of Cardiology and is validated both for in-hospital and 6-month mortality [29]–[31]. There is also an alternative GRACE risk score developed to assess the risk after discharge to six months [32]. This score, however, fails to evaluate in-hospital deaths. An appropriate number of points were given for age, heart rate, systolic blood pressure, creatinine plasma concentration, Killip class, and cardiac arrest (all parameters assessed on admission). All patients were also scored for ST-segment deviation and elevated cardiac markers. Next, all subjects were divided into a high-risk group (≥155 points) and a non-high-risk group, according to a previously evaluated clinical cut-off level [33]. All patients were treated according to contemporary guidelines.

Blood samples were collected in EDTA tubes, treated with a commercial DNA extraction kit (Blood Mini, A&A Biotechnology) and stored at −20 degrees Celsius. The SNPs (rs10757278, rs1333049, rs4977574) were assessed with a TaqMan SNP Genotyping Assay on the ABI 7500 real time PCR platform (Applied Biosystems), according to the manufacturer’s instructions. Ten percent of the samples were genotyped twice (quality control requirements).

The analyzed end-point was total long-term mortality. Data concerning survival was retrieved from the local population registry run by a Government Office, assuring the most complete follow-up possible. Target follow-up time was 5 years in the case of the derivation group and 3 years for the validation sample.

A statistical analysis was performed with STATISTICA 9.0 software. Distribution of variables was tested with Shapiro-Wilk test. Next, clinical parameters were compared between the genotypes with chi-2 or Kruskal-Wallis ANOVA test, as appropriate. Survival was compared with a log-rank test. Univariate and multivariate analyses for 5-year survival were performed with a Cox proportional hazards model (derivation group only) with adjustment for sex and age. Variables with significant association with survival were included in a primary model of multivariate regression. In the case of the 3 SNPs, due to the strong linkage between them, we chose rs10757278 to include in the analysis. The final model was selected in a backward stepwise manner. Additionally, all SNPs in the univariate analysis as well as the multivariate model were adjusted for the severity of coronary artery disease (the number of vessels with significant stenosis). Correlation between the GRACE risk score and genotype was tested with Spearman’s rank correlation test. Two-sided p value<0.05 was considered statistically significant. In the case of survival analysis (chi-2 and log-rank tests) multiple tests were performed, therefore p values were adjusted for Bonferroni correction (2 subgroup analyses). Due to very strong linkage between the 3 SNPs, we did not consider them for further multiple testing. The biostatistical parameters were calculated using ARLEQUIN v.3.0 software.

The study was designed to have a statistical power of at least 80 percent to detect a 66% percent relative risk increase in 5-year mortality of high-risk homozygotes compared to other genotypes. Assuming an 18% overall mortality rate and percentage of high-risk homozygotes around 25% [2], the target of events would be achieved in a group of 570 patients. Estimation of sample sizes or effect sizes in survival functions were performed with chi-square test.

## Results

The registry comprised 609 patients. Nine of them were lost to follow-up (1.5%) and genotype could not be determined in 11 remaining cases due to poor sample quality (1.8%). No genotyping discrepancies were observed in the samples genotyped twice. The final derivation group consisted of 589 patients: 25.3% female (n = 149), mean age 62.4±12.0 years, TIMI 3 obtained in 92% of patients (n = 542).

Genotyping results are presented in table 1. The genotype frequency distributions showed no significant deviations from the Hardy-Weinberg equilibrium (rs1333049 SNP: p = 0.12; rs10757278: p = 0.21; rs4977574: p = 0.17). The specific allele frequencies are comparable to previous reports (2, 4, 5). According to genetic localization data, the SNPs rs1333049, rs10757278, and rs4977574 are closely linked. Consequently, pairwise comparison using exact analysis of test disequilibrium yielded departures from independence for all pairs of loci (p*<*0.0001, table 2). We have chosen the rs10757278 SNP to present the clinical characteristics of the study group (table 3). No significant differences were observed between the genotypes. The GRACE risk score showed no correlation with genotype either as a discrete variable (r = −0.028, p = 0.499) or as a variable categorized to 3 risk groups (r = −0.036, p = 0.38).

**Table 1:** Percentages of specific genotypes and associated mortality rates.

Polymorphism(risk allele for MI)	rs1333049 (C)			rs10757278 (G)			rs4977574 (G)		
The whole study group (n = 589)									
Genotype	GG	CG	CC	AA	AG	GG	AA	AG	GG
Percentage (n)	24.8 (146)	46.7 (275)	28.5 (168)	24.2 (143)	47.4 (279)	28.3 (167)	24.1 (142)	47.0 (277)	28.9 (170)
5-year mortality (n)	14.4 (21)	20.0 (55)	13.1 (22)	15.4 (22)	19.4 (54)	13.2 (22)	16.2 (23)	19.5 (54)	12.4 (21)
**Subgroup of high-risk patients (GRACE risk score ≥155, n = 238)**									
**Genotype**	**GG**	**CG**	**CC**	**AA**	**AG**	**GG**	**AA**	**AG**	**GG**
Percentage (n)	26.9 (64)	45.8 (109)	27.3 (65)	26.5 (63)	45 (107)	28.6 (68)	25.2 (60)	47.5 (113)	27.3 (65)
5-year mortality (n)	23.4 (15)^1^	36.7 (40)	13.8 (9)	25.4 (16)^2^	35.5 (38)	14.7 (10)	28.3 (17)^3^	34.5 (39)	12.3 (8)

1 p = 0.007; ^2^p = 0.019; ^3^p = 0.011; chi-2 tests for 3 genotypes; all p values adjusted for Bonferroni correction.

**Table 2:** Linkage disequilibrium of investigated SNPs.

p	SNP1	SNP2	D’	LD	r2
<0.0001	rs1333049	rs10757278	0.88	0.21	0.77
<0.0001	rs1333049	rs4977574	0.83	0.2	0.68
<0.0001	rs10757278	rs4977574	0.89	0.22	0.78

**Table 3:** Baseline characteristics of the study group based on rs10757278 genotype (risk allele for MI- G).

Characteristic	Overall populationN = 589	rs10757278AA homozygotesN = 143	rs10757278AG genotypeN = 279	rs10757278GG homozygotesN = 167	P
Age (years)	62.4 (12.0)	64.0 (11.5)	62.1 (11.8)	61.5 (12.5)	0.2
Female gender (%)	25.3 (n = 149)	29.4 (n = 42)	23.3 (n = 65)	25.1 (n = 42)	0.39
Body mass index (kg/m^2^)	24.7 (3.7)	24.7 (3.5)	24.7 (3.7)	24.7 (3.8)	0.99
Hypertension (%)	53.3 (n = 314)	53.8 (n = 77)	53.4 (n = 149)	52.7 (n = 88)	0.98
Type 2 diabetes (%)	22.1 (n = 130)	27.3 (n = 39)	21.1 (n = 59)	19.2 (n = 32)	0.2
Previous myocardial infarction (%)	10.9 (n = 64)	7.7 (n = 11)	14 (n = 39)	8.4 (n = 14)	0.07
Systolic blood pressure (mmHg)	138.7 (28.3)	136.8 (27.1)	139.3 (30.7)	139.5 (25.1)	0.76
Heart rate (beats/min)	75.7 (17.8)	76.0 (18.5)	76.2 (17.7)	74.6 (17.4)	0.32
Killip class III or IV (%)	5.9 (n = 35)	4.9 (n = 7)	6.8 (n = 19)	5.4 (n = 9)	0.68
ST-elevation in anterior leads (%)	39.4 (n = 232)	35 (n = 50)	41.2 (n = 115)	40.1 (n = 67)	0.45
TIMI flow grade 3 after procedure (%)	92.0 (n = 542)	93.7 (n = 134)	90 (n = 251)	94 (n = 157)	0.21
Stent implantation (%)	77.1 (n = 454)	78.3 (n = 112)	74.9 (n = 209)	79.6 (n = 133)	0.47
No of vessels with significant stenosis	1.7 (0.8)	1.69 (0.8)	1.68 (0.84)	1.78 (0.8)	0.66
eGFR (ml/min/1.73 m^2^)	79.9 (23.3)	78.1 (26.4)	79.8 (22.7)	81.8 (21.6)	0.24
Haemoglobin on admission (mg/dl)	13.5 (1.6)	12.8 (1.5)	13.2 (1.6)	13.0 (1.7)	0.15
Ejection fraction (%)	45.9 (9.5)	46.4 (8.4)	45.4 (9.9)	46.4 (9.5)	0.57
GRACE risk score	149 (35)	152.5 (35.3)	149.4 (35)	147.3 (34)	0.38

eGFR- estimated GFR, mean values with standard deviations are given, unless otherwise specified.

**Figure 1:**
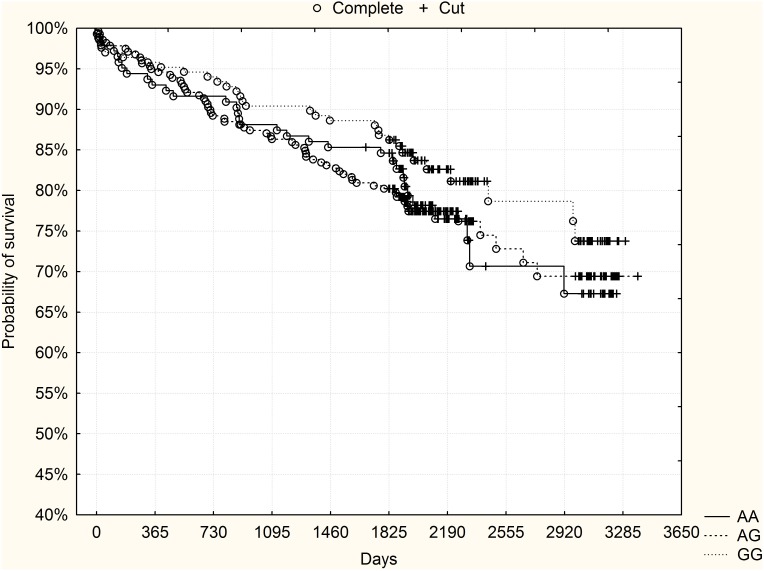
The rs10757278 polymorphism and long-term survival in the derivation group. No significant differences were observed between the genotypes.

A 5-year follow-up with a median of 1950 days (minimum 1824, maximum 3378 days) was performed. At the cut-off point of 1825 days (5 years) 98 patients had died (16.6%). When all the study group was analyzed, no significant differences in mortality were found between the genotypes (table 1). Figure 1 presents Kaplan-Meier surviving curves for specific genotypes of rs10757278 polymorphism and long-term mortality (p = 0.3 after adjustment for Bonferroni correction, log-rank test).

**Figure 2:**
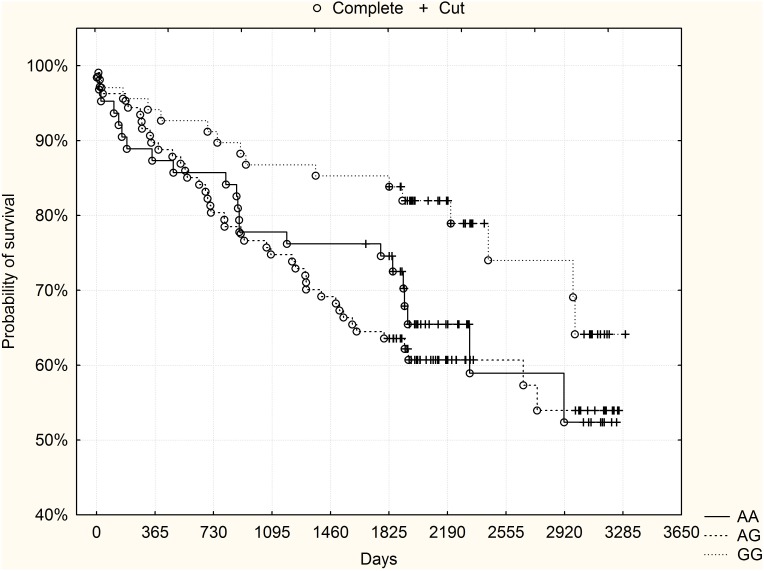
The rs10757278 polymorphism and long-term survival - subgroup of high-risk patients (GRACE risk score ≥155) from the derivation group. GG high-risk homozygotes had significantly higher probability of survival compared to other genotypes (p = 0.032 after adjustment for Bonferroni correction, log-rank test).

However, in the subgroup of high-risk patients (GRACE risk score ≥155, n = 238, 26.9% mortality [n = 64]), visual analysis of survival curves showed strikingly better survival of GG high-risk homozygotes compared to both other genotypes. The curves for heterozygotes and low-risk homozygotes had an almost similar course and were therefore collapsed together for further analysis. Kaplan-Meier surviving curves for the subgroup of high-risk patients and rs10757278 polymorphism are shown in figure 2. The difference was statistically significant (p = 0.032 after adjustment for Bonferroni correction, log-rank test). Mortality rates for specific genotypes in the subgroup of high-risk patients are shown in table 1.

Table 4 presents the results of a Cox proportional hazards model for 5-year survival in a subgroup of high-risk patients. All analyses were adjusted for sex and age. The hazard ratio associated with the high-risk genotype (AA or AG) was: HR = 2.2 (1.15–4.2) for the rs10757278 polymorphism, HR = 2.7 (95% CI 1.3–5.4) for the rs4977574 one and HR = 2.3 (1.2–4.5) for the rs1333049 one. In a univariate analysis, apart from the 3 investigated polymorphisms, significant association was found in the case of Killip class, ejection fraction, and type 2 diabetes. In multivariate regression the rs10757278 polymorphism remained related to mortality together with ejection fraction and type 2 diabetes. After additional adjustment for severity of coronary artery disease all p values were still below 0.05.

**Table 4:** Univariate and multivariate analysis for 5-year mortality in a subgroup of high-risk patients form the derivation group (GRACE risk score ≥155).

Variable	Hazard ratio	95% CI	p
Killip class	1.5	1.2–1.9	0.0007
Ejection fraction (%)	0.95	0.93–0.98	0.00014
Type 2 diabetes	2.1	1.3–3.5	0.0035
rs1333049 GG or CG genotype	2.3 (2.3)	1.2–4.5 (1.2–4.3)	0.015 (0.014)
rs10757278 AA or AG genotype	2.2 (2.2)	1.15–4.2 (1.2–4.2)	0.016 (0.015)
rs4977574 AA or AG genotype	2.7 (2.7)	1.3–5.4 (1.3–5.4)	0.006 (0.006)
No of vessels with significant stenosis	0.98	0.9–1.07	0.6
**Multivariate analysis**			
Ejection fraction (%)	0.96 (0.96)	0.93–0.98 (0.94–0.98)	0.0009 (0.0009)
Type 2 diabetes	2.0 (1.9)	1.2–3.3 (1.2–3.2)	0.009 (0.01)
Rs10757278 AA or AG genotype	2.1 (2.1)	1.1–4.0 (1.1–4.0)	0.026 (0.026)

All values adjusted for sex and age. In the brackets values additionally adjusted for severity of coronary artery disease (number of vessels with significant stenosis) are given.

We validated the results for the rs10757278 SNP in the Warsaw registry population of acute coronary syndrome, which included 365 patients with STEMI. Genotype could not be determined in 10 of the patients (2.7%). The final validation group comprised 355 patients (27.9% of females, n = 99; mean age 63.9±11.6 years; TIMI 3 obtained in 89%, n = 316; table 5). The percentage of GG, AG and AA genotypes was 23.9% (n = 85), 47.6% (n = 169) and 28.4% (n = 101), respectively.

**Table 5:** Baseline characteristics of the validation group based on rs10757278 genotype (risk allele for MI- G).

Characteristic	OverallpopulationN = 355	rs10757278AA homozygotesN = 101	rs10757278AG genotypeN = 169	rs10757278GG homozygotesN = 85	P
Age (years)	63.9 (11.6)	65 (11.7)	64.5 (11.7)	61.2 (10.9)	0.062
Female gender (%)	27.9 (n = 99)	30.7 (n = 31)	27.8 (n = 47)	24.7 (n = 21)	0.66
Hypertension (%)	63.6 (n = 226)	65.3 (n = 66)	62.1 (n = 105)	64.7 (n = 55)	0.84
Type 2 diabetes (%)	20.6 (n = 73)	23.8 (n = 24)	18.9 (n = 32)	20 (n = 17)	0.63
Previous myocardial infarction (%)	13.5 (n = 48)	10.9 (n = 11)	13.0 (n = 22)	17.6 (n = 15)	0.39
Systolic blood pressure (mmHg)	125.7 (21.1)	123.4 (22.7)	126.2 (19.1)	127.4 (22.7)	0.41
Heart rate (beats/min)	79.8 (17.2)	79.8 (18.3)	78.7 (16.2)	82.2 (17.8)	0.28
Killip class III or IV (%)	3.9 (n = 14)	4.0 (n = 4)	3.5 (n = 6)	4.7 (n = 4)	0.9
ST-elevation in anterior leads (%)	44.8 (n = 159)	50.5 (n = 51)	42.6 (n = 72)	42.3 (n = 36)	0.39
TIMI flow grade 3 after procedure (%)	89 (n = 316)	93.1 (n = 94)	87.6 (n = 148)	87 (n = 74)	0.3
Stent implantation (%)	92.4 (n = 328)	92.1 (n = 93)	91.1 (n = 154)	95.3 (n = 81)	0.49
No of vessels with significant stenosis	1.56 (0.73)	1.47 (0.7)	1.59 (0.72)	1.58 (0.8)	0.39
eGFR (ml/min/1.73 m^2^)	56.5 (9.4)	55.5 (11.2)	57.5 (7.8)	55.9 (9.6)	0.48
Ejection fraction (%)	45 (9.2)	44.3 (9.2)	45.5 (9.5)	45.0 (8.7)	0.29
GRACE risk score	156.9 (34.8)	160.7 (35.2)	155.4 (34.9)	155.5 (34.2)	0.42

eGFR- estimated GFR, mean values with standard deviations are given, unless otherwise specified.

**Figure 3:**
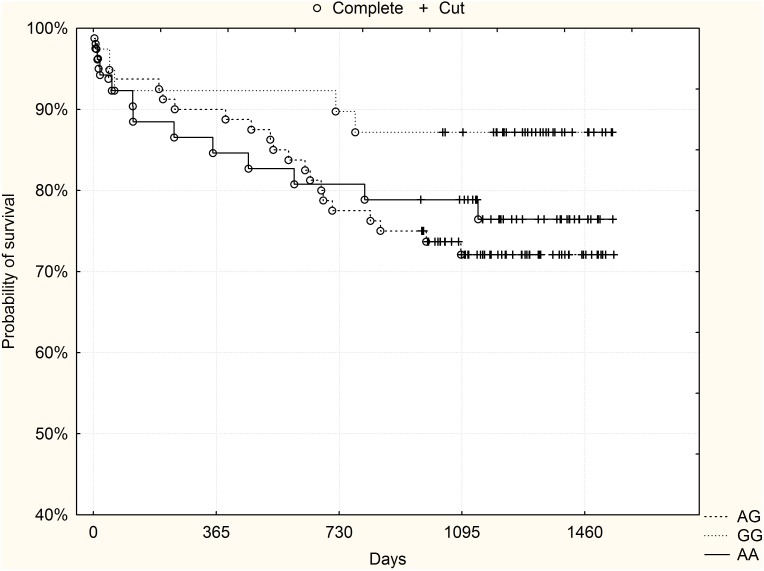
The rs10757278 polymorphism and long-term survival – subgroup of high-risk patients (GRACE risk score ≥155) from the validation group (p = 0.09, log-rank test).

3-year mortality was also analyzed (median follow-up 1312 days, minimum 973 days, maximum 1549 days). Here 55 patients (15.5%) died: 9 GG homozygotes (10.6%), 26 heterozygotes (15.4%) and 20 AA homozygotes (19.8%), (p = 0.21 chi-2 test). High-risk patients according to the GRACE risk score constituted 49.6% of the validation group (n = 176, 54 AA homozygotes, 82 heterozygotes, 40 GG homozygotes). In the subgroup of high-risk patients according to the GRACE risk score, died 5 GG homozygotes (12.5%), 22 heterozygotes (26.8%) and 12 AA homozygotes (22%), (p = 0.2, chi-2 test). In this group, GG homozygotes showed a trend towards a better probability of survival: p = 0.09, log-rank test. Figure 3 shows Kaplan-Meier survival curves for rs10757278 genotypes in the subgroup of high-risk patients from the validation group.

**Figure 4:**
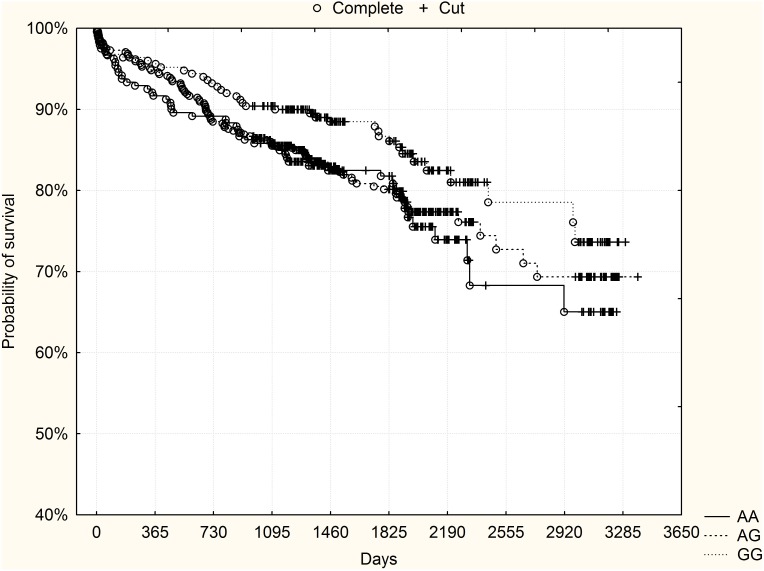
The rs10757278 polymorphism and long-term survival – joint analysis of derivation and validation groups together. GG high-risk homozygotes had significantly higher probability of survival compared to other genotypes (p = 0.04, log-rank test).

**Figure 5:**
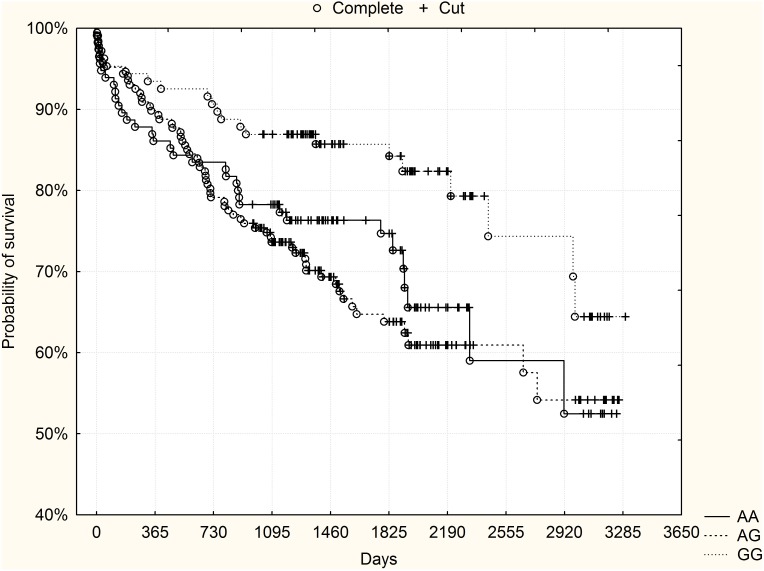
The rs10757278 polymorphism and long-term survival – joint analysis of subgroup of high-risk patients (GRACE risk score ≥155) from derivation and validation groups together. GG high-risk homozygotes had significantly higher probability of survival compared to other genotypes (p = 0.003, log-rank test).

**Figure 6:**
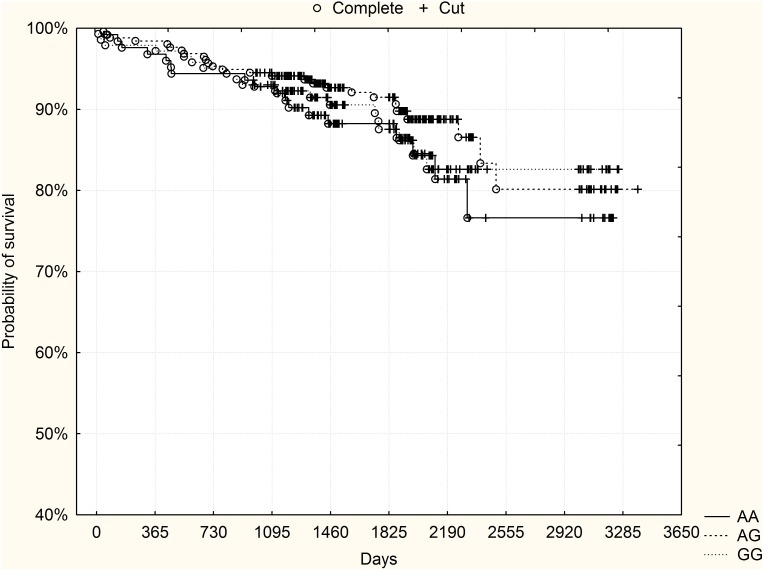
The rs10757278 polymorphism and long-term survival – joint analysis of subgroup of non-high-risk patients (GRACE risk score <155) from derivation and validation groups together. No significant differences were observed between the genotypes.

Figures 4–6 present the results of joint analysis of both derivation and validation groups. There was a significant difference in the probability of survival, with a preferable outcome for GG homozygotes (p = 0.04, log-rank test). Subgroup analysis based on GRACE risk score stratification, however, showed that this finding was only due to a difference observed in high-risk patients (p = 0.003, log-rank test). No clear trend in the course of survival curves was found in non-high-risk patients (p = 0.7, log-rank test).

## Discussion

The 9p21.3 locus showed a significant association with the 5-year survival of high-risk patients with STEMI (GRACE risk score ≥155 points). This phenomenon is probably related to an increased number of events in the chosen subgroup and was observed neither in medium-risk, nor in low-risk patients. Surprisingly, the genotype associated with increased risk for myocardial infarction showed a protective effect in terms of further prognosis, instead of deteriorating it, as reported in some studies as well as in the previous retracted version of this article [28]. Our new findings are additionally confirmed by results from the validation group.

This survey supports several recent studies that also report such a paradox [25]–[27]. In the INFORM Cohort (patients with acute coronary syndromes) the region within the 9p21.3 locus that includes the rs10757278 SNP was significantly associated with the outcome in whites [26]. No such effect took place in other ethnic groups (blacks or Hispanics). The risk genotypes for myocardial infarction were associated with a lower rate of all-cause deaths and cardiac hospitalizations (HR = 0.72, p = 0.0085). Next, this paradox was also reported for patients from Emory Biobank who were undergoing elective coronary angiography. GG homozygotes for the rs10757278 SNP (risk genotype for MI) showed a trend towards better survival and lower MI incidence in a 2.8-year follow-up [27]. Finally, Horne B.D. et al. reported decreased risk for incident MI in patients with coronary artery disease per rs2383206 risk allele [25]. In those studies no subgroup analysis based on clinical risk assessment was presented. Even if a paradoxical phenomenon had been reported for whole study groups, additional testing based on risk groups might reveal that it was specific only for high-risk individuals.

On the other hand, our results are opposite to the GRACE registry that was performed in European populations with acute coronary syndrome, and showed significant association between the rs1333049 polymorphism and outcome within 6 months, [22]. In this case, more cardiac deaths and recurrent MIs were reported for C high-risk allele carriers, as assumed.

The results from European registries may be not in concordance with studies performed in other populations [23], [24]. The effect sizes of specific genotypes may be strongly related to genetic background as well as to environmental factors. Therefore it is essential to validate the results of genetic studies in regional populations. In Han Chinese patients with STEMI, no differences were found between the 9p21 locus genotypes in 2-year event-free survival (cardiac death, non-fatal MI, recurrent angina, or heart failure requiring hospitalization), [24]. Similarly, patients from Post-Myocardial Infarction Study (New Zealand, 9-year follow-up) had genotype-dependent variation neither in total mortality nor in hospital admissions due to reinfarctions or heart failure [23].

There is an Italian study that investigates closely linked polymorphism: rs1333040 [34]. It included participants who had early-onset myocardial infarction (<45 years) and underwent coronary angiography without index event coronary revascularization. During a 10-year follow-up, the genotype significantly affected risk of coronary revascularization, but no influence on cardiac death or recurrent myocardial infarctions was observed. In general, the rate of events was low due to young age (5.1%, n = 77) and possibly therefore no effect on survival was shown.

There are several potential hypotheses that might explain our paradoxical findings. For example, numerous patients with high-risk genotypes for MI may die due to their first event; thus, they are underrepresented in case-control studies. This is a limitation of the study design and may lead to information bias and misinterpretation. However, in our case the percentage of patients with prior MI was comparable between the genotypes. On the other hand, the problem has been raised that patients with prior MI may be more aggressively treated and therefore the additional risk associated with a recurrent event can be to some extent diminished. Next, it is assumed that genetic contribution to disease pathogenesis is attenuated with age and is the most substantial for premature cases. But in terms of prognosis, it would be very limiting to investigate only young low-risk individuals due to a very low number of events. Even if sufficiently large sample sizes were collected, expected hazard ratios would be minimal, with no potential use in clinical practice. The effect observed in our study was generally present only in high-risk individuals; the disadvantage of this finding is that it may be strongly biased by co-morbidities. The next issue that might explain discrepancies between the studies is follow-up duration, which was 6 months in the case of the GRACE registry. In our population we included only patients who survived the first 48 hours from MI, and performed a 5-year-long observation. The mechanisms for deaths may therefore differ between these studies. Furthermore, it is reported that the influence of the 9p21 locus on coronary artery disease is rather more associated with the progression of atherosclerosis and burden than with plaque stability and thrombosis [35]–[36]. If this effect has already manifested itself clinically as CAD or MI and has led to introducing appropriate treatment, the mechanisms associating the 9p21 locus and further prognosis may be completely different. The observed influence may be even opposite.

Based on multivariate analysis, 9p21 locus polymorphism added prognostic information to previously established risk factors like ejection fraction and type 2 diabetes. We observed remarkably high effect sizes for the association between high-risk genotypes and 5-year outcome. It makes these novel risk markers potentially more applicable in everyday practice. Furthermore, if only a particular subgroup of patients was to be genotyped, the method would be more cost-effective. Future interventional studies where additional therapeutic actions are applied to high-risk clinical and genetic profiles would help translate this effect into clinical practice.

The association between the genotype and outcome was limited to high-risk patients according to the GRACE risk score. No such effect was found in low or medium-risk patients. We did not investigate in detail, at which the cut-off level of the GRACE risk score for the effect starts, and we used the previously defined and recommended cut-off value of 155 points [33]. Alternatively, if the effect is linear, we might have missed it due to the low event rate in low and medium-risk patients. Nevertheless, our study was not designed and was thus underpowered for a more detailed subgroup analysis.

High-risk patients according to the GRACE risk score constituted around 40% of our derivation cohort and 50% of the validation group. The relatively large percentages of high-risk patients seem surprising; however they are comparable to the report by Goncalves et al. [37], in which 36.7% of patients with non-ST elevation acute coronary syndrome had >133 points while an additional 21.9% had 113–133 points. Compared to that study group, all patients in our analysis were given a additional 28 points for ST-segment elevation. Furthermore, in the study by Goncalves et al. some GRACE risk scores were lowered due to the inclusion of patients with unstable angina who did not receive 14 points for elevated cardiac enzyme level.

We have found no association between the 9p21.3 locus and either participants’ age or severity of coronary artery disease (table 3), which is consistent with the GRACE registry [22]. On the contrary, in the study from New Zealand, high-risk homozygotes (rs1333049 SNP) were significantly younger, compared to other genotypes (60.6 vs. 62.8 years, p = 0.009) [23]. The report from the United States of America showed that the rs10757278 SNP influenced angiographic severity and the progression of coronary artery disease, [35]. Finally, in the Chinese population the rs1333049 SNP contributed to the severity of coronary artery disease, but only in diabetics [36]. The discrepancies might be again explained by different genetic effect sizes depending on a chosen population.

It is surprising that no significant deviation from the Hardy-Weinberg equilibrium was shown in our population. However, the study was not designed to prove the association between the 3 SNPs and myocardial infarction. Such analysis would require significantly larger sample sizes.

The number of patients investigated in this study was relatively low, but in our opinion it was large enough to search for associations of clinical importance. Very large study groups enable finding significant correlations of very small effect sizes that have no further meaning in everyday practice. A further limitation of the study is the retrospective type of analysis; however, the data was collected prospectively.

## Conclusions

The 9p21.3 locus was associated with 5-year mortality in high-risk patients with STEMI. Surprisingly, the genotype associated with increased risk for myocardial infarction showed a protective effect in terms of further prognosis (a previously published and retracted version of the paper inappropriately reported the opposite results due to genotyping errors).

This finding, due to the very high size of the effect, could potentially be applied into clinical practice. However, the optimal set of risk genotypes and appropriate tools for this clinical setting are still to be identified and elaborated upon.
